# Supplementary material for: Large-scale computational discovery and analysis of virus-derived microbial nanocompartments
Source: Nat Commun. 2021 Aug 6;12:4748. doi: 10.1038/s41467-021-25071-y (PMC8346489; doi:10.1038/s41467-021-25071-y)
Supplement: Supplementary file 1 — Supplementary Information [file 41467_2021_25071_MOESM1_ESM.pdf]

Supplementary Information

Large-scale computational discovery and analysis of virus-derived microbial nanocompartments

Michael P. Andreas[1] and Tobias W. Giessen[1,2]*

[1]Department of Biomedical Engineering, University of Michigan Medical School, Ann Arbor, MI, USA

[2]Department of Biological Chemistry, University of Michigan Medical School, Ann Arbor, MI, USA

Table of Contents

1. Additional data and analysis for Family 1 Encapsulins 2
2. Additional data and analysis for Family 2 Encapsulins 4
3. Additional data and analysis for Family 3 Encapsulins 9
4. Additional data and analysis for Family 4 Encapsulins 13
5. References 14

1. Additional data and analysis for Family 1 Encapsulins

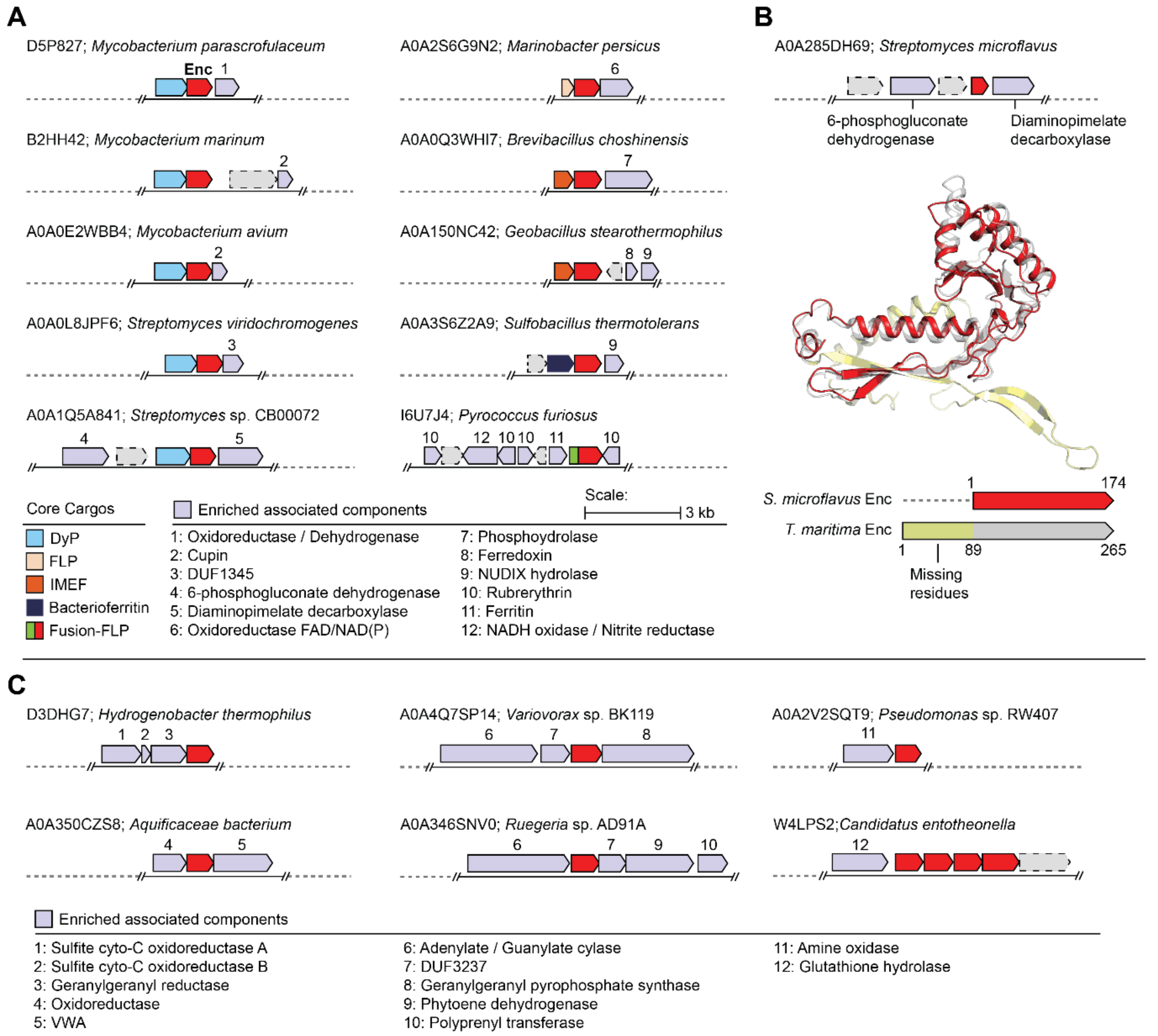

Fig. S1. Family 1 variant and minor operon types. **A)** Operons containing Family 1 encapsulins (red) are illustrated for 5 of the major Family 1 classes (Dyp - light blue, FLP - beige, IMEF - orange, Bacterioferritin - dark blue, FLP-fusion- red and green). Various enriched components (purple) are commonly found in operons for respective cargo classes. **B)** A novel class of short encapsulin identified in *Streptomyces* is characterized by an N-terminal truncation that results in the loss of the N-terminal helix, E-loop, and part of the P-domain. An I-TASSER [1] model (red) was generated using a representative protein sequence (Uniprot: A0A285DH69) from *Streptomyces microflavus*. Alignment with the structure of *T. maritima* encapsulin (PDB: 3DKT) (yellow) indicates the conserved components of the short encapsulins are structurally similar to other Family 1 encapsulins. **C)** Selected Family 1 operons classified as “Minor” that cannot be grouped within the conventional Family 1 cargo classes. These operons do not have clearly defined cargos or targeting peptide sequences, but often contain generally enriched components (purple).

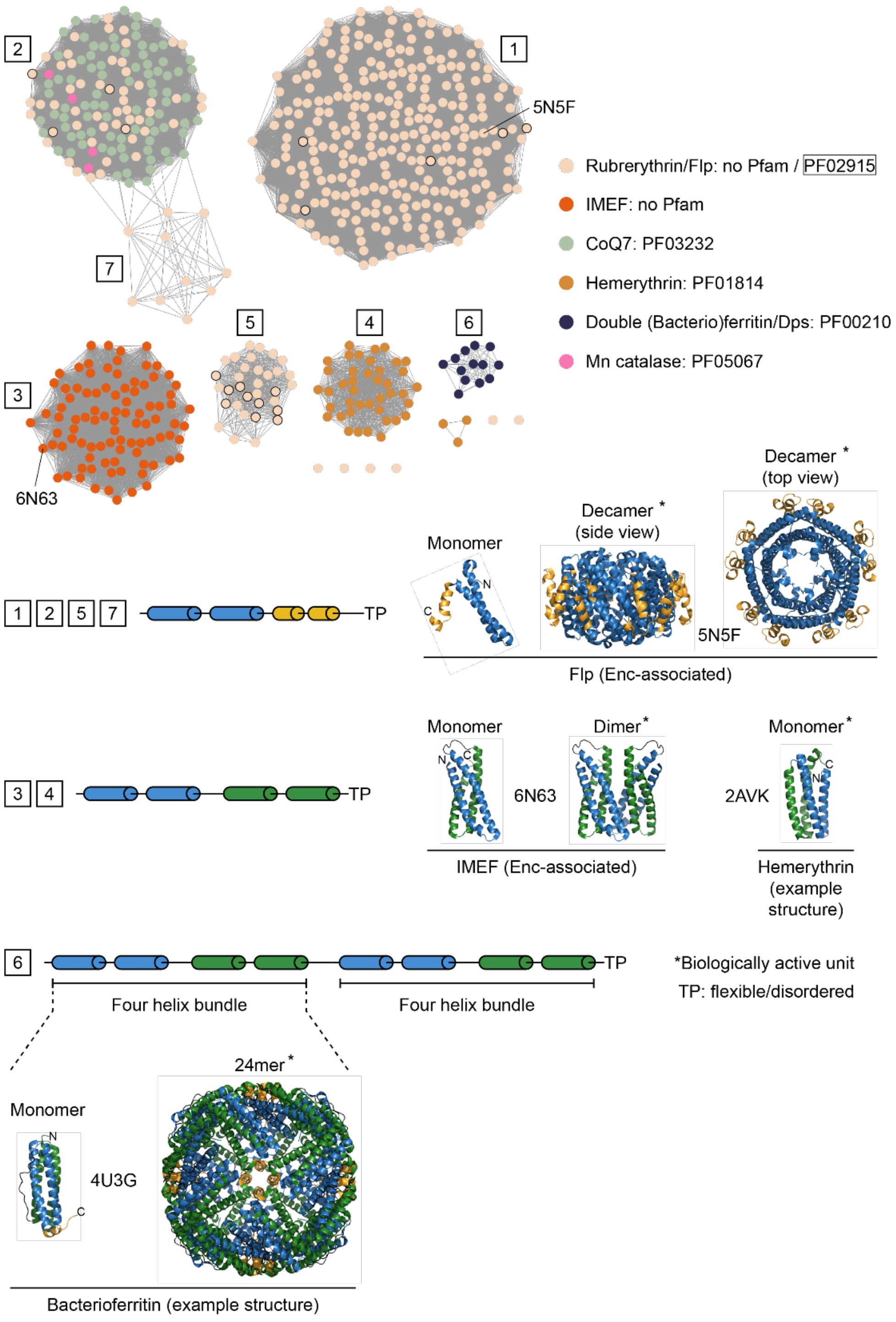

Fig. S2. Ferritin-like protein (Flp) superfamily cargo proteins. Top: SSN of all identified Family 1 Flp cargo proteins. Clusters are numbered from the largest to the smallest cluster. Names and Pfam families are shown on the right. PDB IDs of structurally characterized proteins are indicated. Bottom: Secondary structures of different clusters as analysed by Jpred 4.[2] Example monomer and biologically active unit structures are shown. No encapsulin-associated hemerythrin and bacterioferritin cargo structures are available, thus, general examples are shown (PDB IDs: 2AVK and 4U3G). Cluster 6 contains unusual double four helix bundle proteins that might assemble into 12mer instead of 24mer bacterioferritin-like complexes. TP: targeting peptide.

2. Additional data and analysis for Family 2 Encapsulins

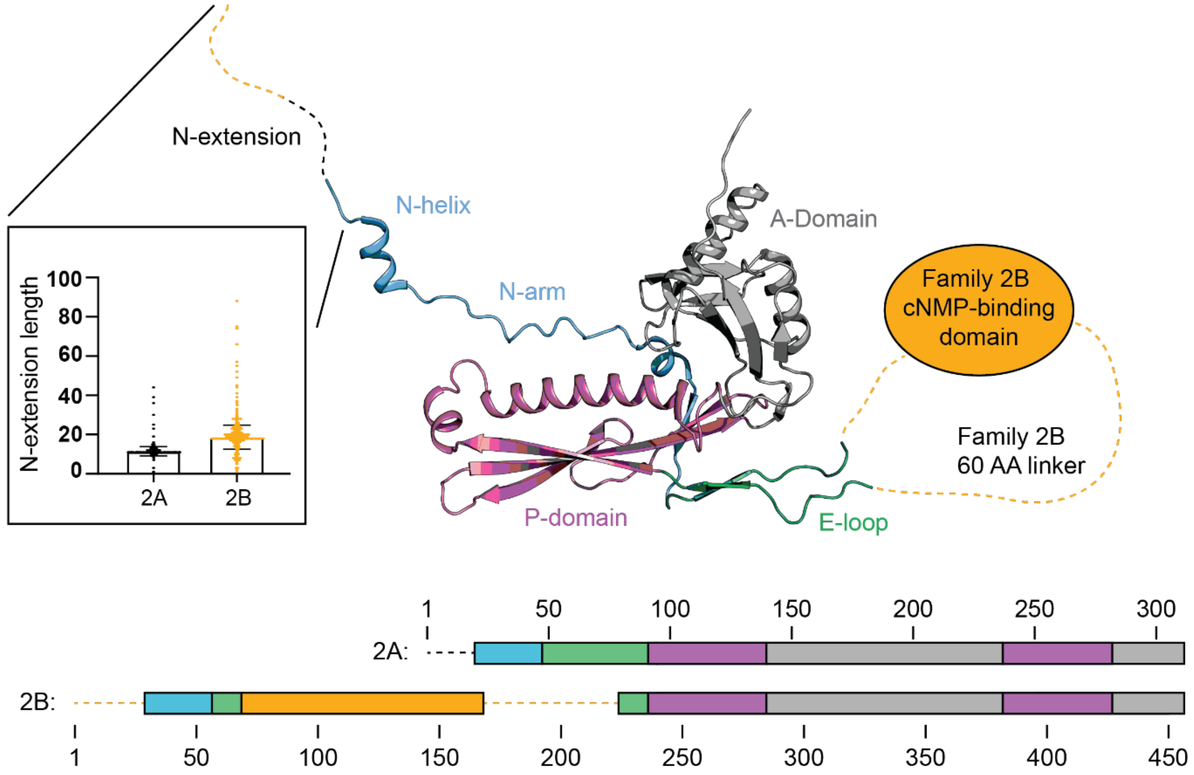

Fig. S3. Structural overview of Family 2 encapsulins. The structure presented here (PDB:6X8M) illustrates the conserved domains between Family 2 encapsulins, namely the N-arm, E-loop, P-domain, and A-domain. Family 2B encapsulins have a cNMP-binding domain and 60 amino acid long linker inserted within the E-loop. This feature is not present in Family 2A encapsulins. As shown by the inset graph, the N-terminal extension found in Family 2B encapsulins is generally longer than in 2A (N-extension lengths: Family 2A- 12 ± 2 AA's; Family 2B- 19 ± 6 AA's). Inset graph was made using GraphPad Prism V 9.0.2. n=74 (2A) and n=163 (2B) sequences were used to create the graph. Data are presented as mean values +/- SD.

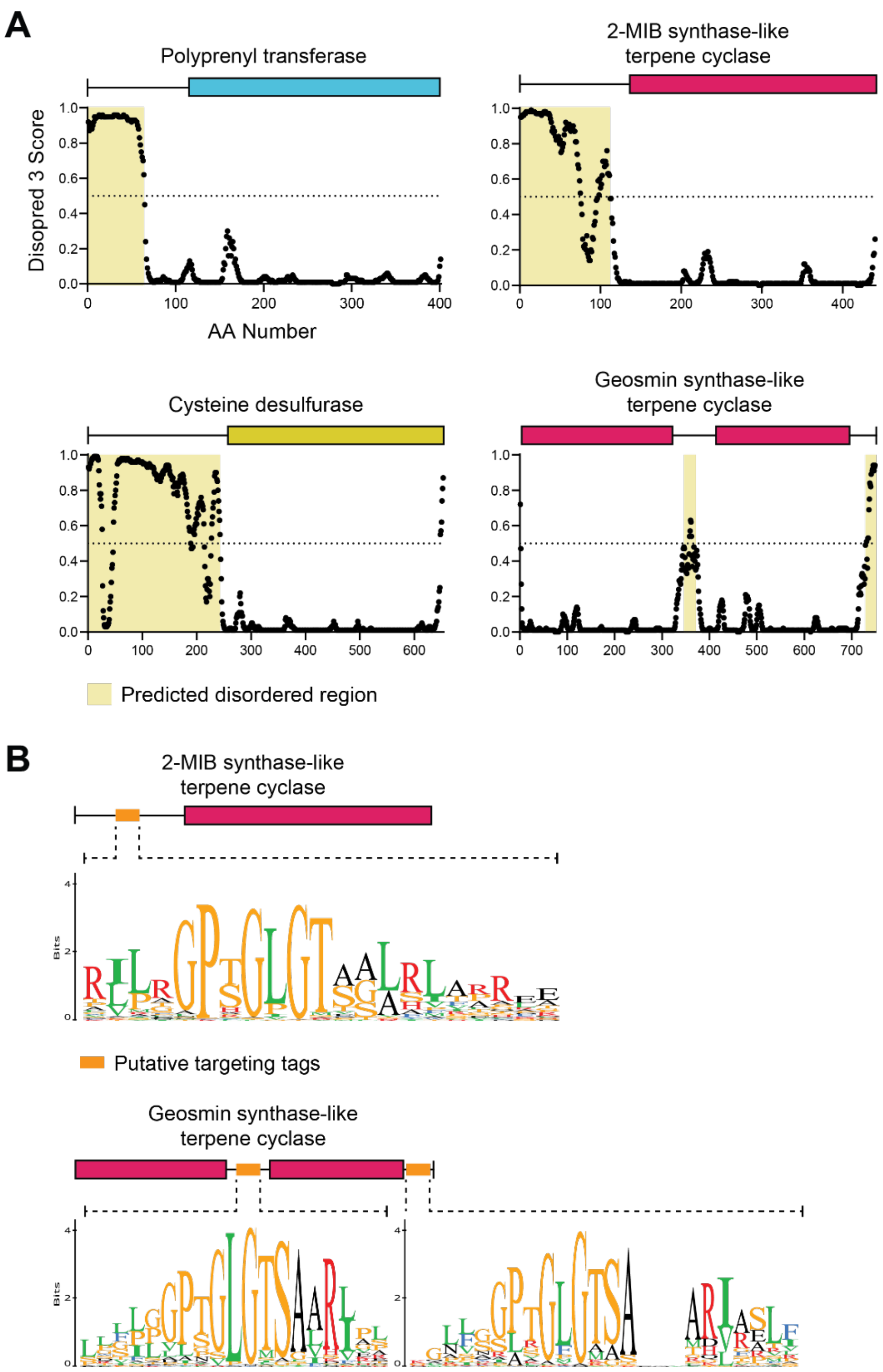

Fig. S4. A) The N-termini of PTs, CDs, and 2-MIB synthase-like TCs are predicted to be disordered. Graphs illustrate per-residue disorder predictions from Disopred3 [3] of representative sequences (CD: A0A010WJT9, PT: A0A0B5EUR5, TC-2MIBS-Like: Q9F1Y6, TC-GMS-Like: A0A3D0QW52). Residues with predicted score of 0.5 or greater (dotted line) are designated as disordered. Regions of disorder are highlighted in yellow. Plots were made using GraphPad Prism V 9.0.2. **B)** Sequence alignments (Clustal Omega V 1.2.2 in Geneious Prime V 2020.1.2) of 499 2-MIB synthase-like TCs show an N-terminal consensus sequence of GPTGLGT. Similar consensus sequences of GPTGLGTSAAR were identified within internal and C-terminal disordered regions from an alignment of 118 Geosmin synthase-like TCs.

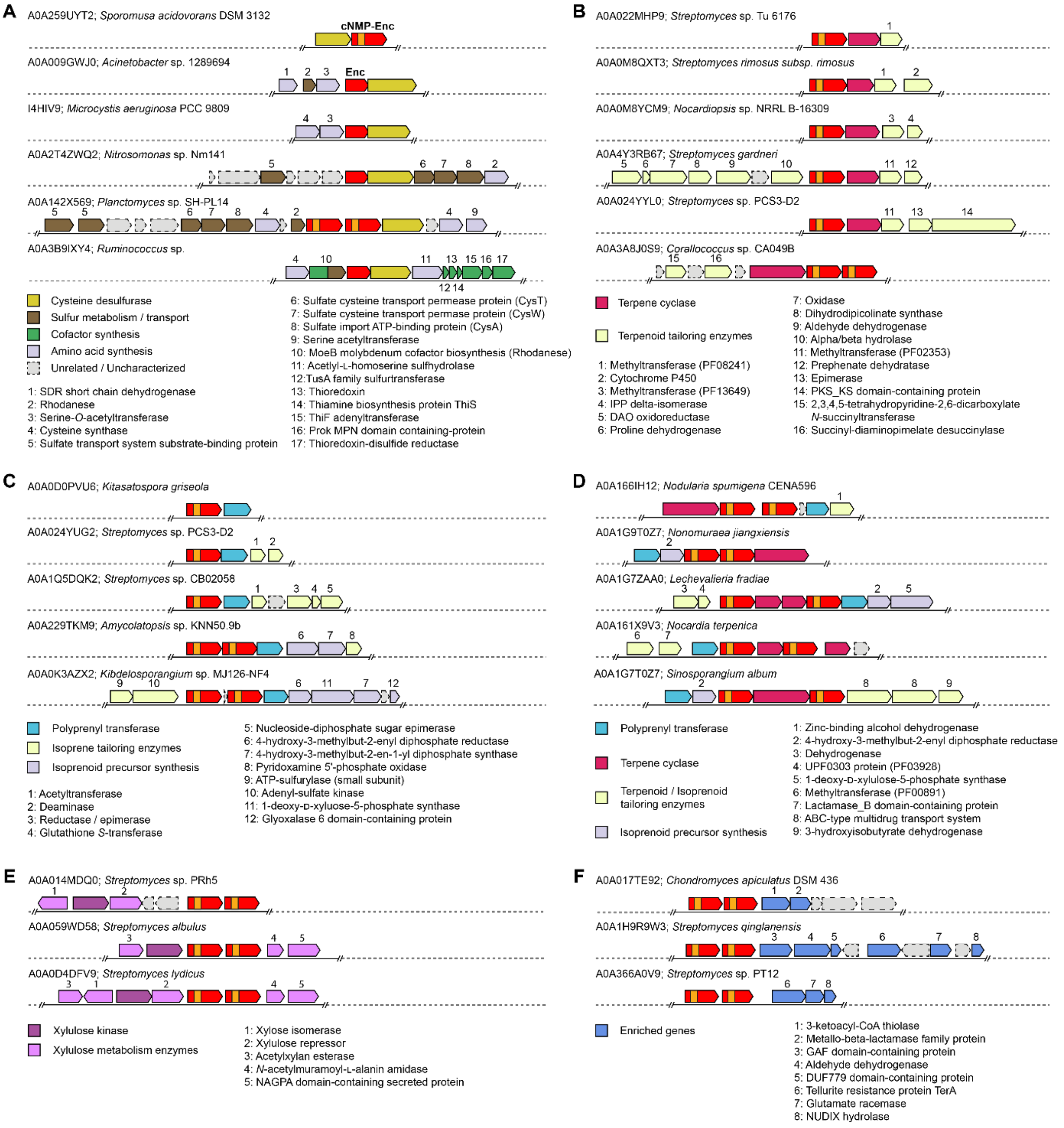

Fig. S5. Family 2 variant and minor operon types. **A)** Selected CD operons containing both Family 2A and Family 2B encapsulins often contain other enriched components that are found in sulfur metabolism and transport (brown), cofactor synthesis (green), and amino acid synthesis (light purple). Serine-*O*-acetyltransferases and rhodaneses are among the most commonly enriched genes in Family 2A operons. **B)** Family 2B TC (pink) operons contain various terpene tailoring enzymes (light yellow). The most commonly enriched tailoring enzymes in TC systems are methyltransferases, but other enriched tailoring enzymes likely expand the diversity of terpenoids produced from these operons. **C)** Family 2B PT (light blue) operons contain enriched tailoring enzymes (light yellow) and enzymes associated with isoprenoid precursor synthesis (light purple). **D)** 165 Family 2A encapsulins were found in operons containing both PTs (blue) and TCs (pink). Similar to PT and TC operons, these mixed operons often contain enriched terpenoid and isoprenoid tailoring enzymes (light yellow) and isoprenoid precursor synthesis enzymes (light purple). **E)** Family 2B XK (dark purple) operons have highly conserved components associated with xylulose metabolism (light purple). It is worth noting that all XK operons encode for two different Family 2B encapsulins. **F)** Minor operons contain enriched components (blue) and do not contain genes encoding for CDs, TCs, PTs, or XKs.

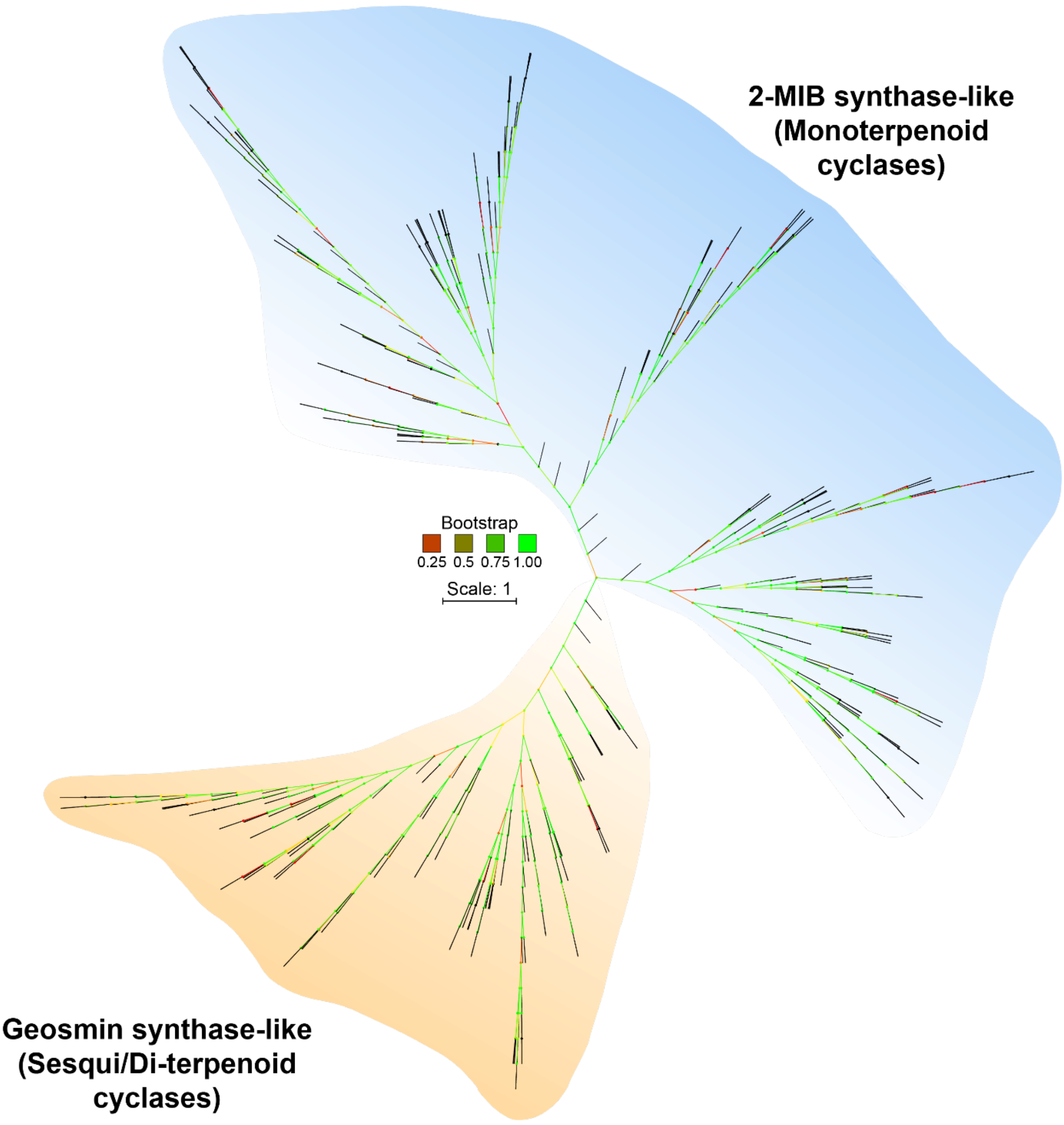

Fig. S6. Phylogenetic analysis of 530 Family 2-associated putative terpene cyclase cargo proteins. MAFFT was utilized for multiple sequence alignments with standard parameters; alignment curation was done via BMGE and standard parameters; for tree inference, PhyML+SMS was employed. Based on homology to characterized enzymes, Family 2-associated terpene cyclases can be divided into two major groups, one similar to geosmin synthase, likely representing sesqui- and diterpenoid cyclases, and one similar to 2-MIB synthase, likely representing monoterpenoid cyclases.

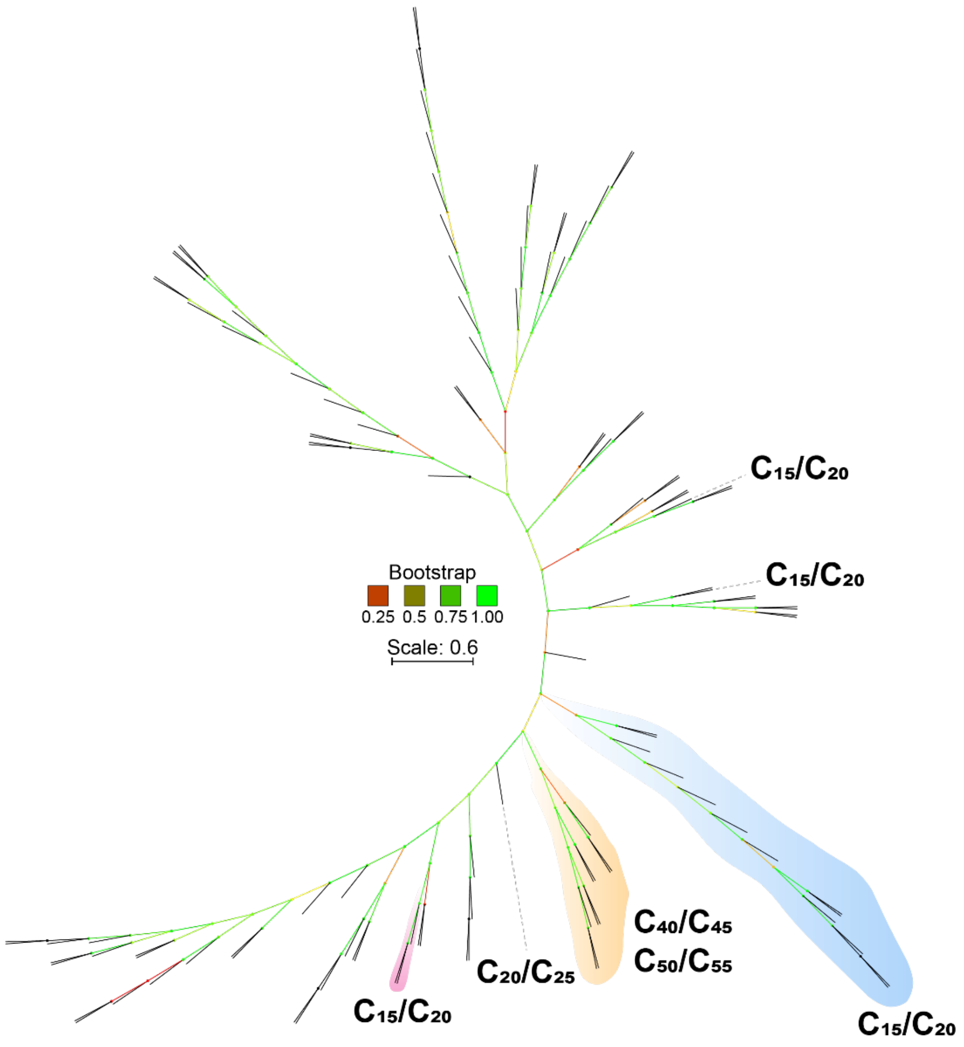

Fig. S7. Phylogenetic analysis of 122 Family 2-associated putative polyprenyl transferase (PT) cargo proteins. MAFFT was utilized for multiple sequence alignments with standard parameters; alignment curation was done via BMGE and standard parameters; for tree inference, PhyML+SMS was employed. 25 characterized PTs were included and are highlighted in color. Beginning with dimethylallyl diphosphate (DMAPP), a series of polyprenyl diphosphates are assembled by PTs. Their product range in terms of the length of the synthesized isoprenoid chain is shown in bold. The following nomenclature indicates products with the respective number of carbon atoms in the linear polyprenyl chain: C_{15}: farnesyl diphosphate, FPP, C_{20}: geranylgeranyl diphosphate, GGPP, C_{25}: farnesylgeranyl diphosphate, FGPP, $C_{40/45/50/55}$: polyprenyl diphosphates.

3. Additional data and analysis for Family 3 Encapsulins

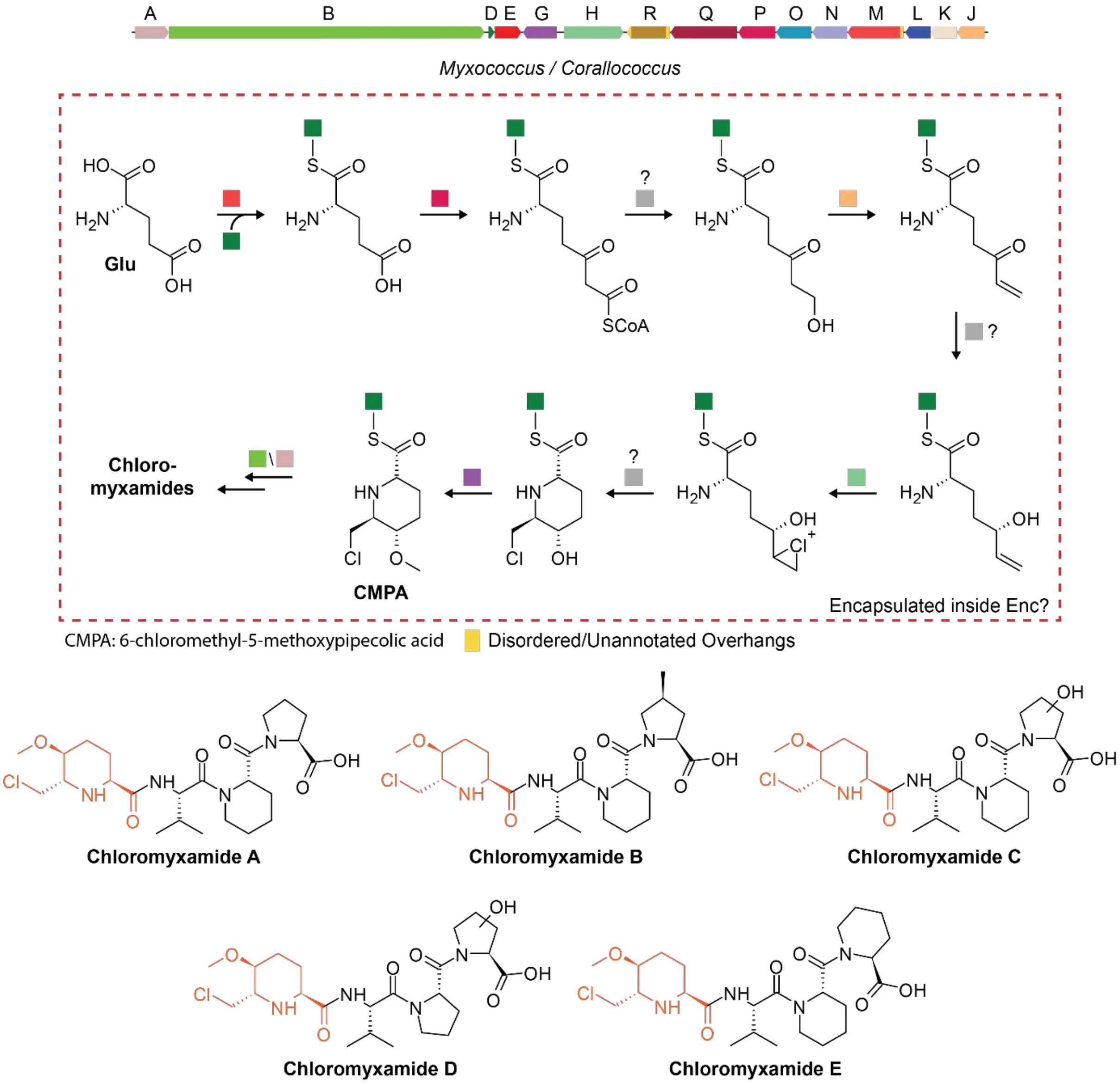

Fig. S8. Top: Family 3 encapsulin-containing chloromyxamide biosynthetic gene cluster and partial proposed biosynthetic route.[4] It is proposed that the CMPA building block is assembled in an amino carrier group-dependent manner. Some or all of the depicted reactions may happen inside a Family 3 encapsulin shell. Disordered and unannotated N-/C-terminal regions of operon components are highlighted yellow and may be involved in mediating cargo loading. The chemical logic behind encapsulation may be the sequestration or protection of reactive or toxic aldehyde/ketone or chlorination intermediates. A: ornithine cyclodeaminase, B: hybrid non-ribosomal peptide synthetase/type I polyketide synthase, D: LysW, E: Family 3 encapsulin, G: SAM-dependent methyltransferase, H: rubber oxygenase A, R: aldehyde dehydrogenase, Q: acyl-CoA dehydrogenase, P; acetyl-CoA acetyltransferase, O: acyl-CoA dehydrogenase, N: acyl-CoA dehydrogenase, M: AMP-dependent synthetase, L: NADP-dependent oxidoreductase, K: TetR/AcrR family transcriptional regulator, J: enoyl-CoA hydratase. Bottom: family of chloromyxamides biosynthesized by and isolated from *Myxococcus* sp. MCy10608. The chlorinated CMPA building block is highlighted.

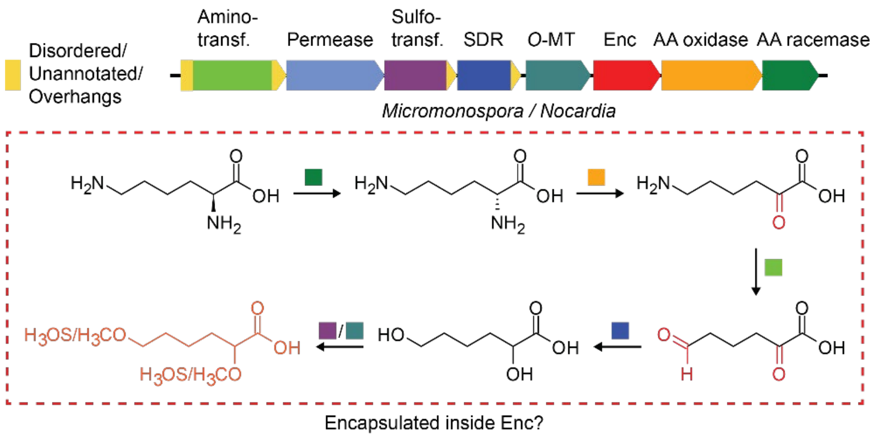

Fig. S9. Top: Family 3 encapsulin-containing natural product BGC found in *Micromonospora* and *Nocardia* species. Disordered and unannotated N-/C-terminal regions of operon components are highlighted yellow and may be involved in mediating cargo loading. SDR: short-chain dehydrogenase/reductase, MT: methyltransferase, AA: amino acid. Bottom: Proposed biosynthetic pathway encoded by the operon shown above. Some or all the steps shown may happen inside an assembled encapsulin shell. The goal may be to sequester reactive/toxic aldehyde/ketone intermediates which are highlighted red.

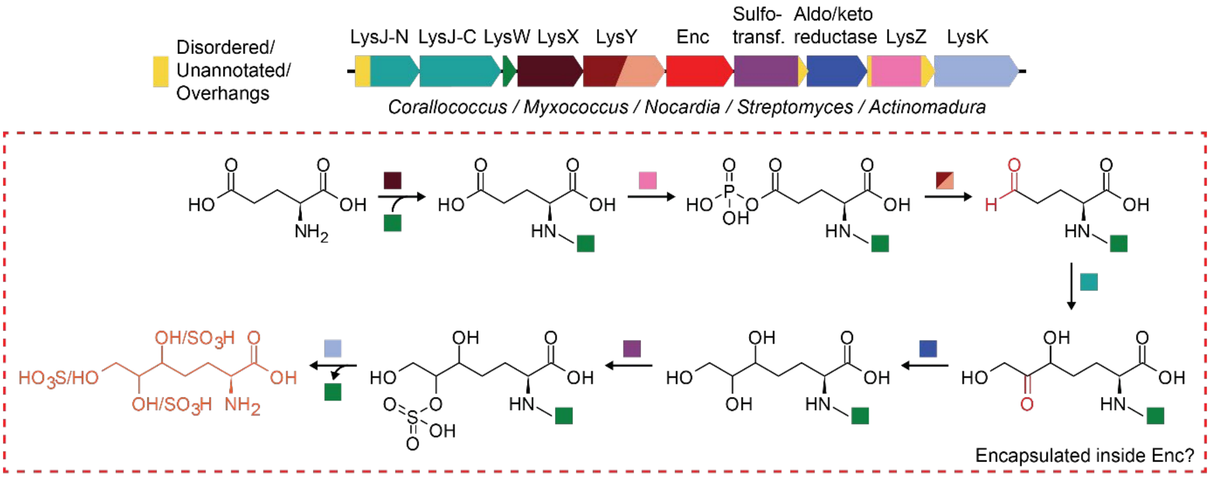

LysJ-N/C: Transketolase LysK: AmCP-AA carboxypeptidase LysW: Amino group carrier protein (AmCP)

LysY: AmCP-AA phosphate reductase LysX: AA-AmCP ligase LysZ: AmCP-AA kinase

Fig. S10. Top: Family 3 encapsulin-containing natural product BGC found in Actinobacteria and Proteobacteria. Disordered and unannotated N-/C-terminal regions of operon components are highlighted yellow and may be involved in mediating cargo loading. Bottom: Proposed biosynthetic pathway encoded by the operon shown above. This pathway seems to rely on an amino group carrier protein to covalently tether pathway intermediates. Some or all the steps shown may happen inside an assembled encapsulin shell. The goal may be to sequester reactive/toxic aldehyde/ketone intermediates which are highlighted red.

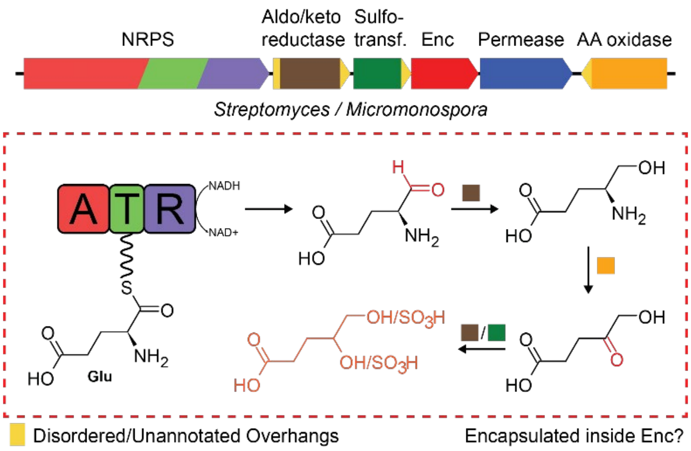

Fig. S11. Top: Family 3 encapsulin embedded in a non-ribosomal peptide synthetase-dependent biosynthetic gene cluster. Disordered and unannotated N-/C-terminal regions of operon components are highlighted yellow and may be involved in mediating cargo loading. Bottom: Proposed biosynthetic route for the gene cluster shown. One or multiple steps may be encapsulated inside a Family 3 encapsulin to potentially sequester reactive or toxic aldehyde/ketone intermediates (red).

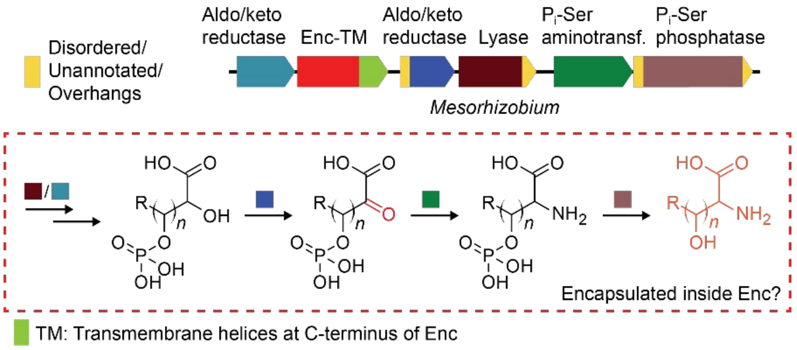

Fig. S12. Top: Gene cluster of an unusual Family 3 encapsulin with a C-terminal fusion of hydrophobic/transmembrane helices. Disordered and unannotated N-/C-terminal regions of operon components are highlighted yellow and may be involved in mediating cargo loading. Some of the steps shown might be encapsulated inside this unusual Family 3 encapsulin.

Family 3 Natural Product encapsulin systems in *Mesorhizobia*:

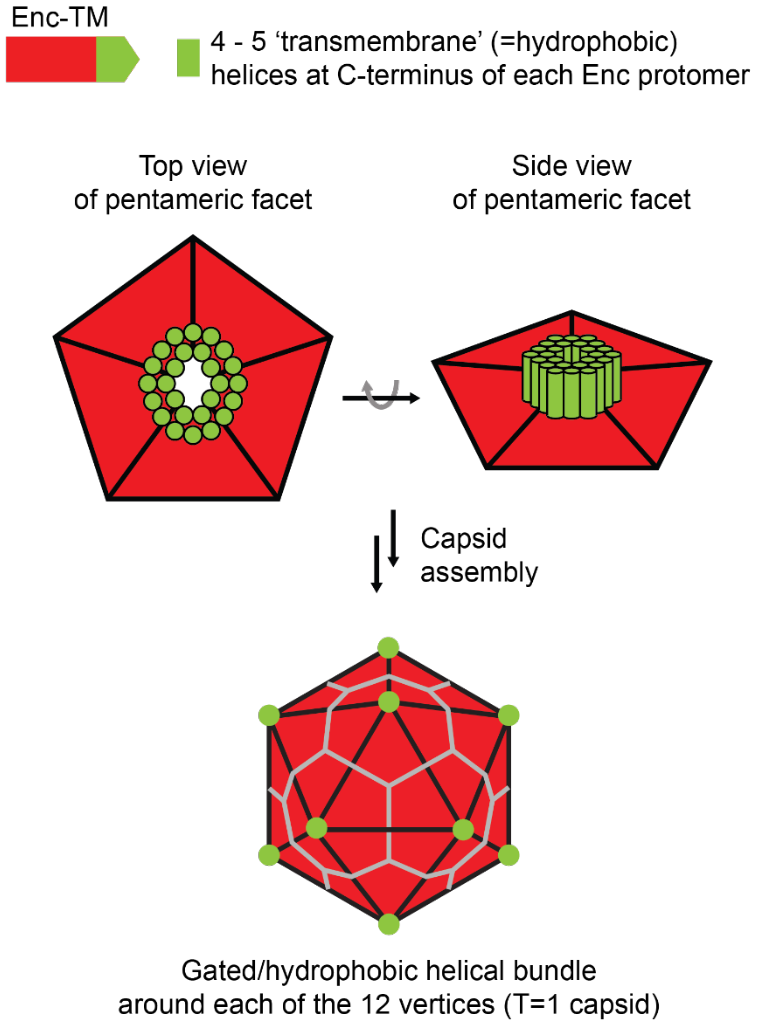

Fig. S13. Special type of Family 3 system found in *Mesorhizobia* species. The conserved HK97-fold encapsulin capsid is C-terminally fused to a 4-5 helix bundle annotated as a major facilitator superfamily (MFS)-type transmembrane protein. Based on the HK97-fold, the C-terminus of the capsid protein is displayed on the outside of the capsid in proximity to the 5-fold symmetry axis/pore. Assuming T=1 icosahedral assembly in the simplest case, this would lead to 5 of these transmembrane or hydrophobic helical bundles meeting at the 12 edges of the icosahedron. This may lead to the formation of a larger all helix pore-like structure surrounding each 5-fold pore. Assuming this helical bundle behaves in a similar fashion to MFS-type membrane proteins, it is conceivable that these helical bundles sitting atop the pores might act as gates controlling the transport of specific, presumably hydrophobic reactants in or out of the encapsulin shell. This of course assumes that topologically, these helical bundles are inverted compared to MFS transporters, i.e. they are hydrophobic on the inside and hydrophilic on the outside. However, if the helical bundles are hydrophobic on the outside, the same way MFS-type proteins are, it might be conceivable that they are able to directly interact with lipid membranes, potentially anchoring the encapsulin close to or even within a membrane or allow the encapsulin to pass through membranes. A final possibility would be that in analogy to viral envelops, this encapsulin system might be surrounded by a lipid membrane, recruited by the 12 hydrophobic helical bundles displayed at its vertices. It should be noted that we assumed T=1 for simplicity's sake and that it is entirely possible that these system may display triangulation number of T=3 or T=4.

4. Additional data and analysis for Family 4 Encapsulins

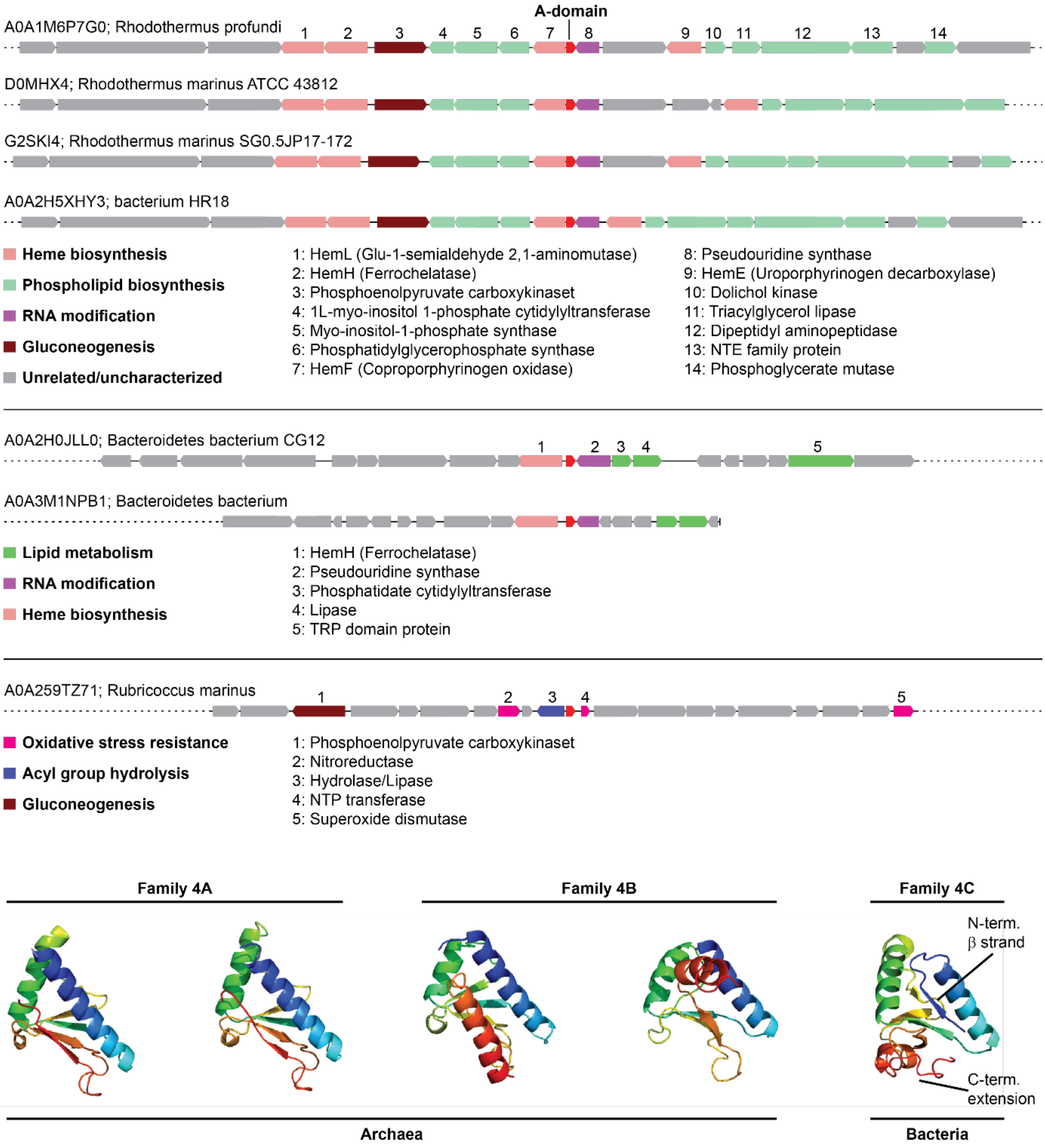

Fig. S14. Top: Bacterial Family 4 operons. A-domain Encapsulins are not part of an obvious operon structure in the sense of transcription direction making it difficult to propose a putative function. The functional categories of proteins encoded in proximity to bacterial A-domain Encapsulins are highlighted. In particular, heme biosynthesis components seem to be the most abundant components. Bottom: Structural comparison of Family 4A, B and C (bacterial) encapsulins.

5. References

1 Yang, J. & Zhang, Y. I-TASSER server: new development for protein structure and function predictions. *Nucleic Acids Res* **43**, W174-181, doi:10.1093/nar/gkv342 (2015).

2 Drozdetskiy, A., Cole, C., Procter, J. & Barton, G. J. JPred4: a protein secondary structure prediction server. *Nucleic Acids Res* **43**, W389-394, doi:10.1093/nar/gkv332 (2015).

3 Jones, D. T. & Cozzetto, D. DISOPRED3: precise disordered region predictions with annotated protein-binding activity. *Bioinformatics* **31**, 857-863, doi:10.1093/bioinformatics/btu744 (2015).

4 Gorges, J. *et al.* Structure, Total Synthesis, and Biosynthesis of Chloromyxamides: Myxobacterial Tetrapeptides Featuring an Uncommon 6-Chloromethyl-5-methoxypipecolic Acid Building Block. *Angew Chem Int Ed Engl* **57**, 14270-14275, doi:10.1002/anie.201808028 (2018).
