# Supplementary material for: Large-scale computational discovery and analysis of virus-derived microbial nanocompartments
Source: Nat Commun. 2021 Aug 6;12:4748. doi: 10.1038/s41467-021-25071-y (PMC8346489; doi:10.1038/s41467-021-25071-y)
Supplement: Supplementary file 2 — Description of Additional Supplementary Files [file 41467_2021_25071_MOESM2_ESM.pdf]

Description of Additional Supplementary Files

File Name: Supplementary Data 1

Description: An annotated and curated spreadsheet of all identified encapsulins and cargo proteins.

File Name: Supplementary Data 2

Description: Annotated sequence similarity networks for each of the four identified encapsulin families in xggml format.
